# Biopriming-Induced Transcriptomic Memory Enhances Cadmium Tolerance in the Cd Hyperaccumulator *Silene sendtneri*

**DOI:** 10.3390/plants15020257

**Published:** 2026-01-14

**Authors:** Mirel Subašić, Alisa Selović, Sabina Dahija, Arnela Demir, Jelena Samardžić, Andrea Bonomo, Gabriele Rigano, Domenico Giosa, Erna Karalija

**Affiliations:** 1 Faculty of Forestry, University of Sarajevo, Zagrebačka 20, 71 000 Sarajevo, Bosnia and Herzegovina; m.subasic@sfsa.unsa.ba; 2 Department of Chemistry, Faculty of Science, University of Sarajevo, Zmaja od Bosne 33-35, 71 000 Sarajevo, Bosnia and Herzegovina; alisa.selovic@pmf.unsa.ba; 3 Department of Biology, Faculty of Science, University of Sarajevo, Zmaja od Bosne 33-35, 71 000 Sarajevo, Bosnia and Herzegovina; sabina.dahija@pmf.unsa.ba (S.D.); arnela.demir@pmf.unsa.ba (A.D.); 4 Institute of Molecular Genetics and Genetic Engineering, Department of Microbiology and Plant Biology, University of Belgrade, Vojvode Stepe 444a, 11042 Belgrad, Serbia; jelena.samardzic@imgge.bg.ac.rs; 5 Department of Mathematics, University of Pavia, 27100 Pavia, Italy; andrea.bonomo02@universitadipavia.it; 6 Department of Biology, University of Rome Tor Vergata, Via della Ricerca Scientifica 1, 00133 Rome, Italy; gabriele.rigano@unitn.it; 7 Program in Space Science and Technology, University of Trento, 38122 Trento, Italy; 8 Department of Chemical, Biological, Pharmaceutical, and Evnironmental Science, University of Messina, 98122 Messina, Italy

**Keywords:** *Silene sendtneri*, biopriming, cadmium tolerance, transcriptomic memory, hyperaccumulator

## Abstract

Seed biopriming is increasingly recognized as a strategy capable of inducing molecular memory that enhances plant performance under heavy-metal stress. Here, we investigated how biopriming *Silene sendtneri* seeds with *Paraburkholderia phytofirmans* PsJN establishes a transcriptional state that predisposes seedlings for improved cadmium (Cd) tolerance. RNA-seq profiling revealed that primed seeds exhibited differential gene expression prior to Cd exposure, with strong upregulation of detoxification enzymes, antioxidant machinery, metal transporters, photosynthetic stabilizers, and osmoprotectant biosynthetic genes. Enrichment of gene ontology categories related to metal ion detoxification, redox homeostasis, phenylpropanoid metabolism, and cell wall organization indicated that biopriming imprints a preparatory transcriptional signature resembling early stress responses. Upon Cd exposure, primed plants displayed enhanced physiological performance, including preserved integrity, elevated antioxidant activity, particularly peroxidases in roots, higher osmolyte accumulation, stabilized micronutrient levels, and substantially increased Cd uptake and sequestration. These coordinated responses demonstrate that biopriming induces a sustained molecular memory that accelerates and strengthens downstream defense activation. These findings demonstrate that PGPR-based biopriming establishes a stable transcriptomic memory in seeds that enhances cadmium tolerance, metal sequestration, and stress resilience, highlighting its potential for improving hyperaccumulator performance in phytoremediation and stress adaptation strategies.

## 1. Introduction

Cadmium (Cd) is a non-essential, highly toxic and mobile heavy metal that increasingly contaminates agricultural soils due to mining, smelting, industrial emissions and long-term use of phosphate fertilizers. Its high bioavailability and efficient transfer into the food chain make Cd one of the most hazardous trace elements for both plants and human health [1]. At the plant level, Cd^2+^ interferes with nutrient uptake and water relations, impairs photosynthesis and respiration, and triggers oxidative stress by excessive production of reactive oxygen species (ROS), ultimately reducing growth and yield [2]. These effects are well documented across crops and medicinal plants, where Cd^2+^ exposure causes chlorosis, root growth inhibition, disruption of membrane and chloroplast ultrastructure, and perturbation of primary and secondary metabolism [3,4]. Therefore, managing Cd^2+^ contamination requires strategies that not only reduce Cd^2+^ entry into the food chain, but also exploit plant species capable of tolerating and sequestering Cd^2+^ for phytoremediation [5].

Among the available approaches, phytoremediation using metal-tolerant or hyperaccumulator species is considered a cost-effective, environmentally friendly strategy for remediating Cd^2+^-polluted soils [6]. Hyperaccumulators combine efficient Cd^2+^ uptake with high translocation to shoots and relatively low toxicity symptoms, offering opportunities for phytoextraction and metal recovery. *Silene sendtneri* is a recently identified Cd^2+^ hyperaccumulator, capable of accumulating high Cd^2+^ concentrations in its aerial tissues while maintaining growth through a combination of organic acid and phenolic metabolism, along with strong antioxidant defenses [7]. Previous studies have shown that seed priming of *S. sendtneri* significantly enhances both Cd^2+^ tolerance and Cd^2+^ accumulation, further improving its potential for phytoextraction. However, the molecular mechanisms by which seed priming induces such long-lasting Cd^2+^ tolerance in this species remain largely unexplored.

Seed priming is a pre-sowing technique in which controlled hydration, often in the presence of chemical or biological agents, initiates early metabolic events of germination without allowing for radicle protrusion. This controlled pre-imbibition accelerates and synchronizes germination, improves seedling establishment and frequently enhances tolerance to subsequent abiotic and biotic stresses [8,9]. Over the past decade, seed priming has gain recognition not only as a physiological treatment, but also as a process that induces stress memory, a persistent “primed state” that enables plants to respond more rapidly or strongly to subsequent stress events [10,11,12,13]. Recent reviews have compiled evidence that priming triggers a range of molecular changes, including enhanced DNA repair, accumulation of signaling proteins and transcription factors, and reconfiguration of hormonal and ROS signaling networks [9,14,15,16,17,18]. These alterations can be maintained through mitotic cell divisions and, in some cases, transmitted to subsequent generations, resulting in within- and trans-generational stress memory [19,20].

At the genomic and chromatin level, stress memory has been associated with changes in histone modifications, DNA methylation and chromatin accessibility at stress-responsive loci, which modulate transcriptional competence upon re-exposure to stress [10,21,22]. Transcriptomic studies in primed plants further support this concept, showing that priming can leave a stable signature of DEGs involved in ROS detoxification, osmoprotection, secondary metabolism and signal transduction, even in the absence of the triggering stress [23,24]. Despite this progress, most mechanistic work of priming has focused on drought, salinity or temperature in model or crop species. Relatively few studies have examined seed priming effects in plants under heavy metal stress, and even fewer have combined transcriptomic analyses with detailed physiological characterization in hyperaccumulators [25].

Among the different priming methods, biopriming, seed treatment with plant growth-promoting microorganisms, has emerged as a particularly promising approach for sustainable stress management [26]. Biopriming with plant-growth-promoting rhizobacteria (PGPR) allows bacteria to colonize the seed surface and internal tissues during imbibition, facilitating early establishment in the rhizosphere and endosphere after germination. This early association can improve seedling vigor and stress tolerance through multiple mechanisms, including production of phytohormones, 1-aminocyclopropane-1-carboxylate deaminase (ACC) deaminase, siderophores, exopolysaccharides, and induction of systemic resistance [27]. Numerous studies show that PGPR-based biopriming can enhance plant tolerance to heavy metals by modulating metal availability, improving nutrient acquisition and strengthening antioxidant defenses [10,27,28]. Metal-tolerant PGPR have been successfully used to increase biomass and metal uptake in hyperaccumulator plants, thereby improving phytoremediation efficiency and reducing stress symptoms [10]. In wheat and other crops, biopriming with rhizobacteria has been shown to enhance Zn and other metal uptake, mitigate oxidative damage, and improve yield under contaminated conditions [28]. The extent to which these beneficial effects arise from durable transcriptomic “pre-conditioning” at the seed stage remains poorly understood.

Species of the genus *Paraburkholderia* have attracted particular attention as versatile PGPR capable of enhancing plant growth, nutrient use efficiency and stress resilience across a wide range of hosts [29]. Although originally isolated from spruce, *Paraburkholderia phytofirmans* PsJN is a well-characterized broad host-range plant growth-promoting bacterium that has been successfully used in seed priming and stress tolerance studies across diverse herbaceous plant species. *Paraburkholderia phytofirmans* PsJN, is a well-characterized endophytic strain that promotes plant growth and confers tolerance to heat, cold, drought and salinity, and has recently been shown to alleviate heavy metal stress. Biopriming *Solanum lycopersicum* ‘Micro Tom’ seeds with PsJN improved growth and photosynthetic performance under nickel stress and reduced Ni accumulation in fruits, highlighting the potential of PsJN-based priming for safer crop production in metal-contaminated environments [10]. However, there is still limited information on how PsJN-mediated biopriming reshapes seed transcriptomes in hyperaccumulator species and how these changes relate to subsequent tolerance to Cd^2+^.

*Silene sendtneri* has recently emerged as a promising model for Cd^2+^ phytoextraction due to its high Cd^2+^ accumulation capacity, strong tolerance and adaptable growth habit [7]. *Silene sendtneri* is a perennial species widely distributed in metal-rich habitats and characterized by substantial biomass production and strong tolerance to heavy metals. Previous studies have demonstrated its pronounced ability to accumulate cadmium in both roots and shoots while maintaining physiological function, supporting its classification as a Cd^2+^ hyperaccumulator. These traits make *S. sendtneri* a particularly suitable model for investigating seed-level priming effects and stress memory mechanisms in hyperaccumulator plants. Previous work has shown that seed priming further enhances Cd^2+^ accumulation and tolerance in *S. sendtneri*, with organic acids and phenolic compounds playing crucial role in Cd^2+^ detoxification and compartmentation in this species. Alongside its well-documented antioxidant and secondary metabolite responses under Cd^2+^ stress, these characteristics make *S. sendtneri* an excellent system for investigating how biopriming-induced molecular memory intersects with intrinsic hyperaccumulation strategies. Despite the demonstrated phenotypic benefits of priming in *S. sendtneri*, no study so far has integrated seed-level transcriptomic profiling with plant-level physiological and biochemical responses under Cd^2+^ stress in this species. More broadly, transcriptomic “signatures” of biopriming in hyperaccumulator plants remain underexplored, and the potential of PGPR-induced memory to fine-tune metal transport, sequestration and ROS-scavenging networks is still largely hypothetical.

In this study, we address existing gaps by integrating RNA-seq analysis of bioprimed versus non-primed *S. sendtneri* seeds with comprehensive physiological and biochemical characterization of in vitro-grown plants exposed to Cd^2+^. Specifically, we (i) identify gene expression and pathway alterations associated with biopriming-induced transcriptomic memory, focusing on metal homeostasis, ROS detoxification, cell wall remodeling and signaling; and (ii) connect these seed-level molecular signatures to biomass production, pigment content, osmolyte accumulation, antioxidant enzyme activities and mineral homeostasis in Cd^2+^-stressed plants derived from the same primed seeds. By linking biopriming-induced transcriptomic reprogramming with enhanced Cd^2+^ tolerance in this hyperaccumulator species, our study aims to provide mechanistic insights into how PGPR-based priming can be harnessed to create “stress-ready” plants for phytoremediation of Cd^2+^-contaminated environments.

## 2. Results

### 2.1. RNA Seq Experimental Design, Sequencing Metrics and Quality Control of S. sendtneri Seeds

The whole de novo transcriptome was assembled from over 625 million raw paired-end RNA-sequencing reads, represented by over 596 M reads (95.37%) after quality filters and adapter removals. Such amount of data allowed us to reconstruct the first transcriptome of *S. sendtneri* composed of 393,920 distinct RNA sequences, that was found to be surprisingly enriched in non-coding RNAs (283,431 transcripts), representing the 71.95% of the entire transcriptome, followed by 106,576 mRNAs (27%), with 83 putative rRNAs and 194 tRNAs. The remaining 3 636 RNAs remained without hint and were classified as unknown (Supplementary Table S1).

The functional annotation was highly comprehensive, successfully assigning attributes to 91,427 (86.11%) of total protein-coding genes. EggNog and COG databases provided the broadest coverage, characterizing 85.51% and 80.41% of the transcripts, respectively. Within the COG classification, significant biological activity was identified in signal transduction (9.03%) and post-translational modification (7.07%), though a notable portion (21.99%) remained functionally cryptic (S category). The structural domain analysis via PFAM provided further resolution for 56.46% of total mRNAs, ensuring a robust functional landscape for this species (Table 1).

### 2.2. Biopriming Establishes a Distinct Transcriptomic Memory Signature in S. sendtneri Seeds

**Figure 1:**
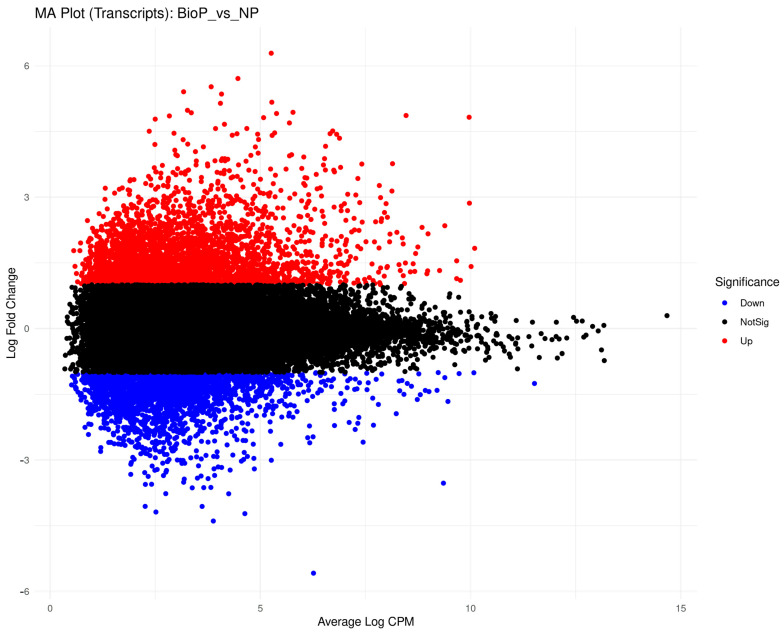
MA plot of differentially expressed genes (DEGs) in bioprimed (BP) vs. non-primed (NP) *S. sendtneri* seeds. DEGs are colored based on gene regulation direction, up (red), down (blue), and not significant (black). The *x*-axis represents mean CPM values, while the *y*-axis represents a log scale of the fold change.

RNA-seq analysis revealed that biopriming of *S. sendtneri* seeds with *Paraburkholderia phytofirmans* PsJN resulted in transcriptional reprogramming before exposure to cadmium stress. An average of 31 M raw reads per sample, of which about 28.6 M good quality after trimming step, were used for investigating DEGs. In total, 39,293 transcripts resulted expressed (TPM > 1) in both experimental conditions, whereas 16,897 and 17,547 were found only in bioprimed (BP) and in nonprimed (NP) samples, respectively, thus resulting in only 19.85% of the total transcriptome found to be expressed. A total of 5509 DEGs, 3188 were up—(2411 mRNA, 773 lncRNA, 3 tRNA and 1 rRNA) and 2320 were down-expressed (1779 mRNA, 537 lncRNA and 5 rRNA) in bioprimed seeds (Figure 1).

**Figure 2:**
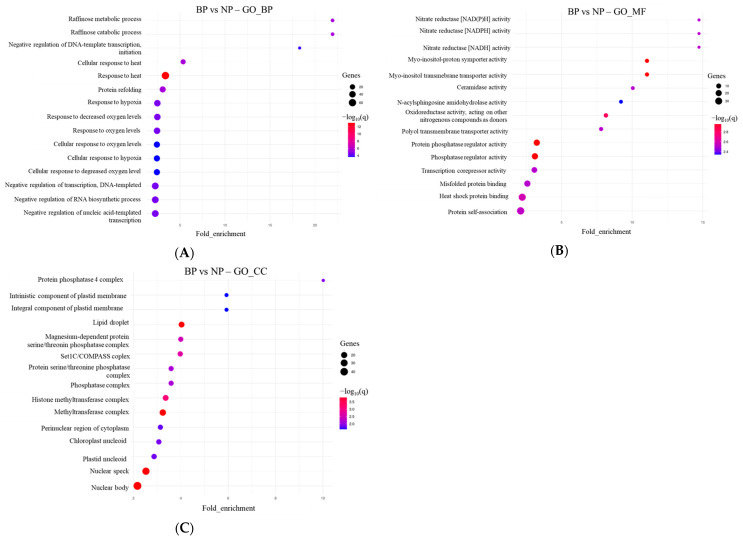
Gene Ontology (GO) enrichment of DEGs in bioprimed (BP) vs. non-primed (NP) *S. sendtneri* seeds. (**A**) Biological Process (GO_BP), (**B**) Molecular Function (GO_MF), and (**C**) Cellular Component (GO_CC) terms significantly enriched in BioP seeds. Bubble size represents the number of genes per category; bubble color indicates −log10 (*q*-value); the *x*-axis shows fold enrichment.

Gene Ontology (GO) enrichment analyses for Biological Processes (GO_BP), Molecular Function (GO_MF) and Cellular Component (GO_CC) pathway further revealed that biopriming upregulated key processes involved in metal ion detoxification, antioxidant metabolism, and structural reinforcement (Figure 2).

GO_BP demonstrated that biopriming significantly upregulated processes associated with metal ion detoxification, oxidative stress management, secondary metabolism and cell wall modification (Figure 2A).

GO Molecular Function (GO_MF) analysis revealed enrichment in metal-binding proteins, ion transporters, antioxidant enzymes, and ABC transporter activities (Figure 2B). BP seeds exhibited increased expression of genes associated with metal ion binding, peroxidase activity, oxidoreductase activity, and transmembrane transporter activity. These functions are directly linked to heavy metal handling and ROS detoxification, indicating that priming enhances the biochemical toolkit available to seedlings during early growth under Cd conditions.

Enriched GO Cellular Component (GO_CC) pointed to a strong representation of genes linked to the cell wall, apoplastic compartments, plasma membrane transport complexes and vacuoles (Figure 2C). These structures are central to Cd^2+^ immobilization, binding, transport and compartmentalization. Increased expression of cell wall- and vacuole-associated genes suggests that priming enhances the structural capacity of seeds and emerging seedlings to restrict or sequester Cd^2+^ once it becomes available in the growth medium.

GO enrichment analysis was used here to provide an overview of functional themes among DEGs; however, given the known limitations of GO annotation (including annotation bias and broad parent terms), mechanistic interpretation was based primarily on targeted, gene-level evaluation of functional families directly linked to metal detoxification, redox homeostasis and transport

### 2.3. Key Biopriming-Responsive Genes Underpin Mechanisms of Cadmium Tolerance

To move beyond broad functional enrichment and to support mechanistic interpretation, we performed a targeted analysis of DEGs belonging to functional groups known to underpin Cd tolerance in plants. This gene-centric approach focused on detoxification/redox maintenance, ROS signaling and scavenging, metal transport and sequestration, osmoprotectant metabolism, and cell wall/phenylpropanoid pathways. Differential expression analysis (log2FC ≥ 1; padj ≤ 0.05) revealed that biopriming activates a distinct set of functional genes whose roles are tightly associated with known mechanisms of heavy-metal tolerance, redox stability, and early stress acclimation. Several high-confidence upregulated genes corresponded to key enzymatic and structural components involved in detoxification, osmoprotection, and cellular homeostasis (Table 2).

The main signature of the biopriming response was the strong induction of glutathione-dependent detoxification genes, including glutathione reductase (chloroplastic) (log2FC > 9) and multiple class III peroxidases, such as Detoxification 27-like peroxidase (log2FC > 6).

Biopriming also markedly upregulated osmoprotectant biosynthetic genes, most notably delta-1-pyrroline-5-carboxylate synthetase (P5CS), the rate-limiting enzyme for proline synthesis. P5CS transcription increased strongly in BP seeds (log2FC > 6), indicating an early metabolic shift toward enhanced osmotic adjustment and ROS scavenging potential. Additional carbohydrate-modifying enzymes, including glycosyltransferases and galactosyltransferases, were also induced, pointing to increased availability of soluble sugars and structural carbohydrates even before Cd^2+^ exposure.

Several metal transport and sequestration genes showed pronounced upregulation. Multiple ATP-binding cassette transporters, including ABC transporter C family member 14-like and ABC transporter G family member 24/31-like (log2FC > 7–10), were strongly induced.

Several ion transporters involved in micronutrient export or mobilization were downregulated in BP seeds (Table 3), including NRAMP3-like metal transporter and multiple Mg^2+^ transporters (NIPA9- and MRS2-type).

Biopriming also enhanced transcripts associated with photosynthetic protection and plastid stability, including several chlorophyll *a*–*b* binding proteins (log2FC > 5–6) and small chloroplastic heat shock proteins (HSP20 family). These genes play roles in protecting photosystems and maintaining pigment integrity under stress.

Numerous regulatory genes were differentially expressed, including transcription factors belonging to WRKY, MYB, NAC, AP2/ERF, bHLH, and bZIP families, as well as redox-associated respiratory burst oxidase homologs (RBOHs) (Table 4).

Pathway analysis was specifically interrogated for pathways relevant to heavy-metal tolerance, including glutathione metabolism, sulfur metabolism, ABC transporters, phenylpropanoid biosynthesis, and plant stress signaling pathways. However, none of these pathways reached statistical significance at the pathway enrichment level, a limitation commonly observed in de novo transcriptomes of non-model plant species where stress responses are distributed across multiple functional modules rather than concentrated in single pathway. To complement broad GO enrichment and reduce dependence on high-level functional categories, we conducted a targeted, gene-family analysis of DEGs central to metal detoxification and stress tolerance; key functional modules and representative genes are summarized in Supplementary Table S1.

### 2.4. Biopriming Enhances Growth and Biomass Production Under Cadmium Stress

**Figure 3:**
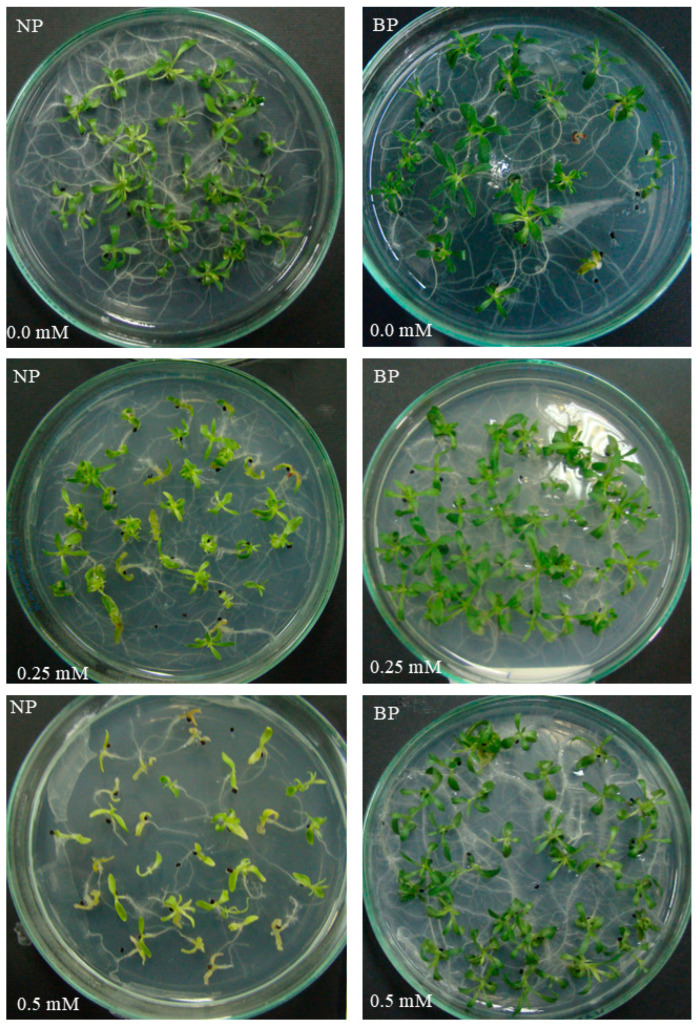
Plant phenotypes of nonprimed (NP) and bioprimed (BP) plants grown under different Cd^2+^ stress levels.

Cadmium exposure reduced growth and biomass in *S. sendtneri*, with nonprimed (NP) plants showing the strongest inhibition (Figure 3). In contrast, bioprimed (BP) plants maintained significantly higher fresh and dry biomass under all Cd concentrations (Table 4; Figure 3). At 0 mM Cd^2+^, BP roots accumulated more fresh biomass than NP roots, suggesting an early priming effect even in the absence of stress. At 0.25 mM Cd^2+^, BP plants exhibited only minor reductions in both shoot and root biomass, whereas NP plants displayed a pronounced decline in shoot FW and DW (Table 5). In case of 0.50 mM Cd^2+^, NP plants failed to develop sufficiently and were excluded from analysis, while BP plants still produced measurable biomass, indicating a stable tolerance phenotype supported by priming-mediated physiological readiness (Figure 3).

Cadmium stress also significantly decreased chlorophyll *a*, chlorophyll *b*, and total chlorophyll in NP plants, reflecting damage to the photosynthetic apparatus (Table 6). BP plants, however, maintained pigment levels comparable to the control at 0.25 mM Cd^2+^. Even at 0.50 mM Cd^2+^, BP plants retained moderate concentrations of chlorophylls, indicating reduced Cd^2+^-induced inhibition of pigment synthesis and degradation. Carotenoid content increased in BP plants under all treatments, with values 1.6×. 4.3× higher than those of NP plants. Since carotenoids are key components of photoprotective machinery, their sustained accumulation suggests that biopriming enhances antioxidant buffering capacity and photochemical stability under Cd stress.

### 2.5. Osmoprotectants Accumulate More Strongly in Bioprimed Plants

Both NP and BP plants exhibited increased proline accumulation in response to Cd^2+^ stress; however, BP shoots displayed higher proline levels across all treatments (Table 7). Proline content in shoots in BP plants was approximately 2–3 times higher than in NP plants under control and 0.25 mM Cd^2+^ conditions, confirming that biopriming pre-activates osmoprotective pathways even before stress exposure. At 0.50 mM Cd^2+^, shoot proline declined in both treatments but remained substantially higher in BP plants, demonstrating maintained metabolic responsiveness under severe stress.

In roots, NP plants accumulated higher baseline proline levels than BP plants. Nevertheless, BP roots showed a gradual and consistent increase with rising Cd^2+^ concentration, whereas NP roots displayed only minor changes.

### 2.6. Primed Plants Exhibit Stronger Antioxidant Responses Under Cd Stress

Cadmium exposure altered antioxidant enzyme activity in *S. sendtneri*, with clear differences between NP and BP plants (Table 8). In BP shoots, CAT activity peaked at 0 mM Cd^2+^ and 0.25 mM Cd^2+^, whereas POD activity was exceptionally high at 0 mM Cd^2+^, indicating a constitutively activated peroxidase-based defense system in primed plants.

In roots, BP plants showed a stronger POD response compared with shoots and NP plants. POD activity in BP roots reached >6000 U mg^−1^ protein min^−1^ at 0.25 mM Cd^2+^, representing the highest antioxidant activity observed across all treatments. CAT activity in BP roots also increased at 0.25 mM Cd^2+^ and remained detectable even at 0.50 mM Cd^2+^, whereas NP did not develop. The predominance of POD activity over CAT under cadmium stress is consistent with the strong transcriptomic induction of multiple class III peroxidases and the known sensitivity of catalase to heavy metal inhibition. These results indicate a peroxidase-centered antioxidant response, particularly in roots, rather than a CAT-dominated detoxification strategy.

### 2.7. Biopriming Improves Mineral Homeostasis in Cd-Exposed Plants

Cadmium stress markedly disrupted mineral homeostasis in NP plants. At 0.25 mM Cd^2+^, Fe and Zn concentrations in NP tissues dropped to roughly one third of their control values (Table 9), indicating strong competition between Cd^2+^ and essential micronutrients for uptake and transport. NP plants exposed to 0.50 μM Cd did not develop sufficiently for mineral analysis, confirming their high Cd sensitivity. In contrast, BP plants maintained substantially higher Fe and Zn levels under Cd exposure. At 0.25 mM Cd^2+^, Fe and Zn concentrations in BP plants remained clearly above those of NP plants at the same Cd level and even exceeded NP controls for Fe. At 0.50 μM Cd^2+^, Fe strongly increased in BP plants, while Zn remained higher than in NP plants at 0.25 μM Cd^2+^, suggesting that priming supports both micronutrient acquisition and internal redistribution under severe stress.

Cd accumulation was consistently higher in BP plants compared with NP plants at 0.25 μM Cd^2+^ and further increased at 0.50 μM Cd^2+^, confirming an enhanced hyperaccumulation phenotype. At 0.25 mM and 0.5 mM Cd, bioprimed plants accumulated high levels of cadmium in whole-plant biomass, consistent with the hyperaccumulating nature of *S. sendtneri* and the high bioavailability of Cd under in vitro conditions.

## 3. Discussion

### 3.1. Biopriming Establishes a Transcriptomic Memory That Pre-Conditions S. sendtneri Seeds for Cadmium Stress

The RNA-seq analysis demonstrates that biopriming with *Paraburkholderia phytofirmans* PsJN imprints a distinct transcriptomic state in *S. sendtneri* seeds before exposure to Cd^2+^. Bioprimed seeds showed strong enrichment of GO categories related to metal ion detoxification, oxidation–reduction processes, response to ROS, cell wall organization and phenylpropanoid biosynthesis, while MF and CC terms pointed to metal-binding proteins, antioxidant enzymes, plasma membrane transport complexes, apoplast and vacuoles. This pattern is highly consistent with the concept of transcription regulation, whereby a priming stimulus induces stable molecular changes that render plants “pre-alerted” to subsequent stress [13,15,21,22].

Most previous work on priming-induced memory has focused on drought, salinity or temperature in model or crop species [13,15,16,19,21,22]. Our data extend this concept to a Cd^2+^ hyperaccumulator and shows that PGPR-based biopriming can pre-activate precisely those pathways that are central to heavy-metal tolerance: glutathione-dependent detoxification, cell wall reinforcement, vacuolar sequestration and ROS management [5,6,12,20,25]. The transcriptional rewiring is evident at the seed stage, long before Cd^2+^ exposure, indicating that *P. phytofirmans* biopriming does not simply accelerate germination but establishes a persistent, functionally relevant memory that shapes subsequent seedling development and performance.

### 3.2. Biopriming-Responsive Genes Provide a Mechanistic Basis for Enhanced Cd Tolerance

The Cd^2+^ concentrations applied in this study (0.25 and 0.50 mM) represent a biologically relevant stress range for hyperaccumulator plants under in vitro conditions and were selected to resolve priming-induced differences in transcriptomic and physiological responses rather than to establish absolute tolerance thresholds. The functional annotation of DEGs in bioprimed seeds reinforces this interpretation. Upregulated genes included chloroplastic glutathione reductase, multiple class III peroxidases (e.g., DETOXIFICATION 27-like), RBOH-like NADPH oxidases, and several WRKY transcription factors, forming a regulatory and enzymatic network poised to buffer redox perturbations and to propagate ROS signals in a controlled manner. The strong induction of delta-1-pyrroline-5-carboxylate synthetase (P5CS) suggests an early metabolic shift towards elevated proline biosynthesis, while glycosyltransferases and related enzymes point to enhanced capacity for soluble sugar accumulation, osmoprotection and cell wall modification.

Equally important is the upregulation of several metal transport and sequestration genes, including ABC transporters (C- and G-family members), a vacuolar cation/H^+^ exchanger (CAX2-like), and Zn transporters. In hyperaccumulators, these components are key for vacuolar storage of metal–thiol complexes and for maintaining micronutrient balance under metal stress [5,6,12,25]. Their pre-activation in seeds implies that seedlings will be able to mobilize Cd^2+^ detoxification and compartmentation pathways more rapidly and efficiently once exposed to Cd^2+^.

In contrast, several genes involved in micronutrient remobilization were downregulated in BP seeds, including an NRAMP3-like metal transporter and multiple Mg^2+^ transporters (MRS2- and NIPA-type). Such repression may help retain essential ions within seed tissues and reduce the risk of Cd^2+^-induced displacement during early germination. Downregulation of selected receptor-like kinases, transporter isoforms and specific peroxidases further indicate fine-tuning rather than uniform up- or downregulation of stress-related pathways. Together, these adjustments create a balanced transcriptional architecture that enhances detoxification capacity while maintaining ion homeostasis and controlled signaling. The DEG landscape in bioprimed seeds provides a mechanistic blueprint for enhanced Cd tolerance, showing how *P. phytofirmans*-induced memory programs seeds and seedlings for faster, stronger and more coordinated responses once Cd^2+^ stress occurs.

### 3.3. Biopriming Helps Maintain Growth and Photosynthetic Performance Under Cd Stress

The seed-level transcriptomic memory translates into a clear physiological advantage under Cd^2+^ stress. Non-primed plants showed pronounced growth inhibition and failed to develop at 0.50 mM Cd^2+^, while bioprimed plants maintained measurable root and shoot biomass even at the highest Cd^2+^ concentration. This shift in the tolerance threshold is particularly relevant for a hyperaccumulator such as *S. sendtneri*, which is inherently Cd^2+^-tolerant [7,12], and indicates that PsJN biopriming can further reinforce intrinsic tolerance traits.

The preservation of chlorophyll *a*, chlorophyll *b* and total chlorophyll in bioprimed plants at 0.25 mM Cd^2+^, and their partial maintenance even at 0.50 mM, suggests that priming protects the photosynthetic apparatus from Cd^2+^-induced damage. Cadmium typically disrupts chloroplast ultrastructure, inhibits photosystem activity and enhances photooxidative stress [3,14]. In our study, bioprimed plants not only retained pigments but also accumulated carotenoids at levels several tens of times higher than those of NP plants. Carotenoids are central to non-photochemical quenching and singlet oxygen scavenging, and their massive induction in BP plants is consistent with a primed photoprotective system. The early upregulation of light-harvesting complex proteins and chloroplastic HSP20 chaperones in seeds provides a plausible molecular underpinning for this improved pigment stability.

These findings support a scenario in which transcriptomic memory established at the seed stage confers a structural and redox “buffer” on chloroplasts, allowing bioprimed seedlings to sustain photosynthetic competence and biomass accumulation under Cd^2+^ stress conditions that are lethal to non-primed plants.

### 3.4. Enhanced Osmoprotection and Antioxidant Capacity in Primed Plants

Osmoprotectant dynamics further support the existence of a primed defense state. Bioprimed shoots exhibited 2–3-fold higher proline levels than non-primed shoots under control and moderate Cd^2+^ exposure, and still maintained elevated proline at 0.50 mM Cd^2+^, when NP plants could not develop. Proline is a multifunctional stress metabolite that contributes to osmotic adjustment, stabilization of proteins and membranes, and scavenging of ROS [16]. Its consistent induction in BP tissues matches the strong upregulation of P5CS in bioprimed seeds and indicates that osmolyte biosynthesis is part of the imprinted memory program.

Differences in root proline patterns between NP and BP plants are also informative. Higher baseline proline in NP roots, coupled with limited change upon Cd^2+^ exposure, suggests a relatively rigid osmotic response. In contrast, the gradual, Cd^2+^-dependent increase in root proline in BP plants points to a more plastic and dynamically regulated adjustment, likely controlled by the primed transcriptional network. Such plasticity may be advantageous under fluctuating metal availability, enabling roots to fine-tune osmotic balance and ROS buffering with minimal metabolic cost.

Antioxidant enzyme activity measurements provide another line of evidence for priming-enhanced defense. In bioprimed shoots and especially roots, POD activity was high, with root POD reaching more than 6000 U mg^−1^ protein min^−1^ at 0.25 mM Cd^2+^. This is in line with the strong induction of class III peroxidases in BP seeds and suggests that *P. phytofirmans* biopriming establishes a peroxidase-centered detoxification system that can be rapidly mobilized upon stress. CAT activity in BP roots increased at moderate Cd^2+^ and remained detectable at 0.50 mM Cd^2+^, whereas NP plants could not survive at that level. Although CAT values in BP shoots were lower than in NP under some conditions, the combination of high POD and robust osmoprotectant accumulation likely provided sufficient ROS detoxification and redox buffering. The enzyme activity patterns observed here are supported by transcriptomic data, which revealed strong upregulation of class III peroxidase genes but no comparable induction of catalase-related transcripts, indicating coordinated regulation at the molecular and biochemical levels.

Similar priming-enhanced antioxidant and osmoprotective responses have been reported in bioprimed cereals and vegetables under drought, salinity and heavy metal stress [8,11,16,17,18,26,27,28]. Our data show that these principles also apply to a Cd^2+^ hyperaccumulator and, crucially, that they are anchored in a clearly defined transcriptomic memory state.

### 3.5. Biopriming Reinforces Mineral Homeostasis and Cd Hyperaccumulation

A key feature of *S. sendtneri* is its ability to hyperaccumulate Cd^2+^ while maintaining physiological function [7,12]. In this context, the combined patterns of Fe, Zn and Cd^2+^ in NP and BP plants are particularly revealing. Non-primed plants suffered strong declines in Fe and Zn at 0.25 mM Cd^2+^, typical of competitive interference between Cd^2+^ and essential micronutrients at uptake and transport sites [3,5,20]. In contrast, bioprimed plants maintained much higher Fe and Zn concentrations under Cd^2+^ exposure, and Fe even increased markedly at 0.50 mM Cd^2+^.

At the same time, Cd^2+^ accumulation was consistently higher in BP plants than in NP plants, with very high Cd^2+^ levels reached at 0.50 mM Cd^2+^. The elevated Fe concentrations observed in bioprimed plants at higher Cd^2+^ exposure occurred alongside preserved growth, pigment stability, and survival, indicating that Fe accumulation was effectively integrated into cellular homeostasis rather than causing oxidative damage via uncontrolled Fenton reaction. The upregulation of ABC transporters, CAX2-like exchangers and Zn transporters, and the downregulation of NRAMP3 and selected vacuolar iron transporters in seeds, dovetail with this interpretation. The strong induction of a vacuolar cation/proton exchanger 2 (CAX2-like) is consistent with enhanced vacuolar handling of divalent cations, which may contribute to cadmium tolerance either through direct sequestration or indirectly via modulation of Ca^2+^ homeostasis and ionic balance [29]. These transcriptional changes are expected to favor vacuolar sequestration of Cd^2+^ complexes and to stabilize Zn and Fe pools, thereby reducing interference between Cd^2+^ and essential cations [5,6,12,25].

This suggests that *P. phytofirmans* biopriming enhances both components of the hyperaccumulator syndrome: tolerance and accumulation. From an applied perspective, such a shift is highly desirable for phytoextraction, where high metal removal from soil must be combined with sustained plant growth [6,7,12,25]. Although bacterial colonization was not directly monitored in this study, evidence from previous experiments using the same priming protocol suggests that *P. phytofirmans* PsJN does not persist as an active endophyte following seed priming. Washing and centrifugation steps remove most exopolysaccharides required for tissue penetration, supporting the interpretation that the observed effects reflect a priming-induced molecular memory rather than sustained bacterial colonization. Recent studies on hyperaccumulator species support the role of PGPR in enhancing cadmium uptake and tolerance; for example, PGPR-mediated hormonal crosstalk and stimulation of lateral root formation significantly increased Cd accumulation in the hyperaccumulator *Sedum alfredii* [30]. Our results suggest that PGPR-based biopriming could be used to “upgrade” hyperaccumulator performance by strengthening ion-homeostatic circuits at the seed stage, before plants encounter contaminated substrates.

Mechanistically, the priming response is supported by DEGs of families with established roles in Cd^2+^ tolerance rather than by GO terms alone. The induction of glutathione-linked redox maintenance (e.g., glutathione reductase and associated redox enzymes) suggests increased capacity to sustain the thiol-dependent detoxification environment required for metal chelation and oxidative stress buffering. Concurrent enrichment of class III peroxidases and RBOH-like genes indicate a primed ROS-signaling/scavenging network. In parallel, strong upregulation of ABC transporters and a CAX2-like exchanger, together with altered expression of Zn transporters and downregulation of NRAMP3-like genes, supports enhanced vacuolar sequestration and re-balancing of micronutrient transport under Cd^2+^ exposure. Transcriptional induction of phenylpropanoid/cell-wall related enzymes is consistent with strengthened structural immobilization capacity. Together, these gene-family level signatures provide a mechanistic basis for the enhanced Cd^2+^ tolerance observed in primed plants. Under in vitro conditions, the absence of soil buffering and sorption results in high metal bioavailability, which can lead to substantial cadmium accumulation in plant tissues, particularly in hyperaccumulator species.

### 3.6. Biopriming Induces Set-Up of a Transcriptional Regulation That Enhances Plant Performance Under Cadmium Stress

Previous studies on *Silene* species have demonstrated strong metal tolerance and accumulation capacities, particularly for Cd^2+^, Zn, and Ni, driven by efficient sequestration, antioxidant defense, and ion homeostasis mechanisms. For example, *Silene vulgaris* and related taxa exhibit enhanced vacuolar metal storage, activation of phenylpropanoid metabolism, and peroxidase-mediated cell wall reinforcement under metal stress. Our findings in *S. sendtneri* are consistent with these reports, while extending them by demonstrating that biopriming establishes a transcriptional memory state that pre-configures these defense pathways prior to stress exposure.

**Figure 4:**
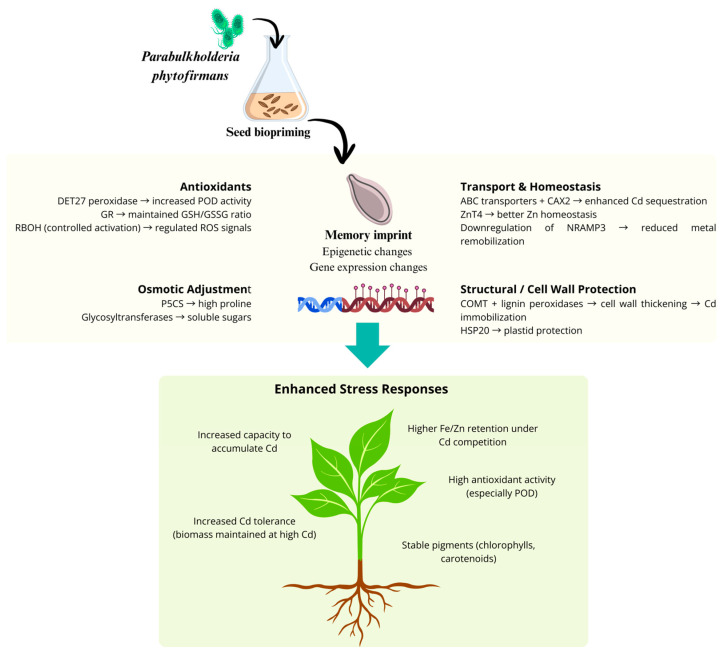
Proposed mechanistic model of biopriming-induced transcriptomic memory and enhanced cadmium (Cd) stress tolerance in *S. sendtneri*.

Our integrated data support a mechanistic model in which biopriming with *P. phytofirmans* establishes a transcriptionally encoded state that prepares *S. sendtneri* for efficient stress mitigation upon Cd^2+^ exposure. This memory is not a transient physiological adjustment but a stable pre-conditioned state that subsequently orchestrates metabolic, antioxidant, and ion-homeostatic responses (Figure 4).

Biopriming of seeds with *P. phytofirmans* triggers a transcriptomic regulation characterized by transcriptional regulation and differential expression of key stress-related genes [31]. Upregulated modules include antioxidant enzymes (DET27 peroxidase, glutathione reductase), ROS-signaling components (RBOH), osmoprotectant biosynthesis genes (P5CS, glycosyltransferases), metal transport and sequestration systems (ABC transporters, CAX2, ZnT4), and structural protection pathways (COMT, lignin peroxidases, HSP20). Downregulated genes such as NRAMP3 reduce metal remobilization, further stabilizing ion homeostasis. These transcriptional changes establish a primed physiological state that enhances antioxidant capacity, osmotic adjustment, Cd^2+^ sequestration, and cell-wall reinforcement. As a result, bioprimed plants exhibit improved Cd^2+^ tolerance, increased Cd^2+^ accumulation, higher Fe/Zn retention under Cd^2+^ competition, and more stable photosynthetic pigments, culminating in an overall enhanced stress-resilient phenotype.

During seed biopriming, the microbial signal from *P. phytofirmans* activates genes involved in peroxidase activity, oxidoreductase processes, glutathione-dependent detoxification, membrane and metal transport, and osmolyte biosynthesis pathways. This early transcriptional reprogramming establishes a distinct molecular memory that persists through germination. As a result, bioprimed seeds enter early development with elevated baseline readiness, allowing the plant to anticipate and more effectively counteract stress before it occurs.

When Cd^2+^ stress is encountered, primed plants activate defense mechanisms more rapidly and intensely than non-primed plants. Photosynthetic pigments remain more stable, minimizing photoinhibition. Osmoprotective metabolism is strongly enhanced, reflected in elevated proline and soluble sugar levels in shoots. Antioxidant systems, particularly POD activity in roots, are massively induced, preventing excessive ROS accumulation. Nutrient uptake systems remain functional in primed plants, limiting the Cd^2+^-induced depletion of Fe and Zn. At the same time, Cd^2+^ uptake and sequestration are enhanced through increased transporter activity and vacuolar binding. These coordinated responses all depend on the prior transcriptional memory established during priming.

Because the defense response is pre-activated, bioprimed plants maintain growth and biomass even under high Cd^2+^ concentrations. Photosynthetic performance is preserved, oxidative system collapse is avoided, and mineral homeostasis remains stable despite competition from Cd^2+^ ions. Primed plants also exhibit enhanced hyperaccumulation capacity, efficiently sequestering Cd^2+^ without compromising essential nutrient status. These traits define a superior tolerance phenotype in which defense, detoxification, and homeostatic processes operate in a metabolically buffered and highly integrated manner.

## 4. Material and Methods

### 4.1. Plant Material and Experimental Design

Seeds of *S. sendtneri* were collected from natural populations in central Bosnia and Herzegovina at Pjeskovita ravan locality (Latitude. 43.91278°; Longitude. 18.46278°; calcareous soil). Seeds were manually cleaned, inspected for uniform size and integrity, and stored dry at 4 °C in darkness until use. All experiments were performed using the same seed lot. This study consisted of two main experimental components:

1. Seed biopriming with *Paraburkholderia phytofirmans* PsJN followed by RNA-seq of bioprimed (BP) and non-primed (NP) seeds, and
2. In vitro cultivation of plants derived from BP and NP seeds under different Cd^2+^ concentrations, followed by assessment of biomass, photosynthetic pigments, osmoprotectants (proline and soluble sugars), antioxidant enzyme activities (CAT and POD) and mineral composition.

Unless otherwise stated, all chemicals were purchased from Sigma-Aldrich (St. Louis, MO, USA), and all solutions and glassware were sterilized prior to use.

### 4.2. Preparation of *Paraburkholderia Phytofirmans* PsJN and Seed Biopriming

*Paraburkholderia phytofirmans* PsJN (DSM 17436) was grown on LB agar plates at 28 °C for 48 h. A single colony was inoculated into 50 mL LB broth (tryptone 10 g L^−1^, yeast extract 5 g L^−1^, NaCl 10 g L^−1^, pH 7.0) and incubated overnight at 28 °C with shaking (150 rpm) until the exponential phase. Cells were harvested by centrifugation (4000 rpm, 15 min), washed twice with sterile distilled water, and resuspended in sterile water. Cell density was adjusted to 10^8^ CFU mL^−1^ using McFarland standards.

For biopriming, seeds were first surface-sterilized by immersion in 70% (*v*/*v*) ethanol for 1 min, followed by 2% (*v*/*v*) NaClO for 10 min, and then rinsed 3–4 times in sterile distilled water. Sterilized seeds were immersed in the *P. phytofirmans* suspension (10^8^ CFU mL^−1^) for 24 h at 4 °C under gentle agitation. After incubation, seeds were briefly treated with 10% (*w*/*v*) Ca(ClO)_2_ for 60 s to remove loosely attached bacteria, rinsed thoroughly with sterile water, dried on sterile filter paper, and stored at 4 °C in sterile microtubes until use. Nonprimed (NP) seeds underwent the same sterilization and imbibition procedure but were incubated in sterile distilled water instead of the bacterial suspension.

### 4.3. In Vitro Germination and Cadmium Treatments

BP and NP seeds were germinated on sterile half-strength Murashige–Skoog (½ MS) medium solidified with 0.8% (*w*/*v*) agar and supplemented with 1% (*w*/*v*) sucrose. Medium pH was adjusted to 5.8 before autoclaving at 121 °C for 20 min. Seeds were germinated and grown in sterile 90 mm Petri dishes containing 25 mL of solidified growth medium. Each Petri dish contained 25–30 seedlings, which were evenly distributed across the surface.

Cadmium was supplied as CdCl_2_·H_2_O (analytical grade) added to the medium, after cooling to <50 °C, to final concentrations of 0 mM Cd^2+^ (control), 0.25 mM Cd^2+^, and 0.50 mM Cd^2+^. Cadmium was and added to the medium after autoclaving to minimize precipitation. Cadmium concentrations refer to nominal values in the growth medium.

Plates were poured under sterile conditions and allowed to solidify. Seeds were placed on the surface of the medium and incubated in a growth chamber under a 16 h light/8 h dark photoperiod, photosynthetically active radiation (PAR) of 120 µmol m^−2^ s^−1^, temperature 23 ± 1 °C and relative humidity 50–60%. Seedlings were harvested after 21 days of growth for all physiological, biochemical and mineral analyses.

### 4.4. Biomass Determination

For each treatment (NP/BP × Cd^2+^ concentration), 20–30 seedlings were randomly selected and separated into shoots and roots. Fresh weight (FW) of shoots and roots was recorded immediately after harvest using an analytical balance. Samples were then dried at 70 °C for 48 h to constant weight and dry weight (DW) was determined. Biomass data are expressed as mg FW or mg DW per plant. Non-primed plants exposed to 0.5 mM Cd^2+^ initiated germination but failed to develop sufficient, viable biomass for reliable biochemical or elemental analysis; therefore, these plants were excluded from quantitative analyses. Representative images are provided for transparency.

### 4.5. Photosynthetic Pigment Quantification

Chlorophyll *a*, chlorophyll *b*, and carotenoid contents were quantified after pigment extraction and expressed per gram of dry tissue (mg g^−1^ DW), independent of per-plant biomass measurements. Photosynthetic pigments (chlorophyll *a*, chlorophyll *b* and carotenoids) were quantified in shoot tissue of *S. sendtneri* seedlings. Dried shoot material (100 mg) was ground to a fine powder in a chilled mortar and pestle. Pigments were extracted with 100% (*v*/*v*) acetone in darkness for 20 min with intermittent vertexing [32]. Extracts were centrifuged at 2000 rpm for 10 min and the supernatant was collected.

Absorbance was measured at 661.6 nm (chlorophyll *a*), 644.8 nm (chlorophyll *b*) and 455.8 nm (carotenoids) using a spectrophotometer. Pigment concentrations were calculated according to Lichtenthaler’s equations [33] and expressed as mg g^−1^ dry weight (DW). All steps were carried out under low light to minimize pigment degradation.

### 4.6. Proline Determination

Free proline content was determined using a ninhydrin-based colorimetric assay according to the modified method of Carillo et al. [34]. Fresh shoot and root tissues (100 mg) were homogenized in 80% (*v*/*v*) ethanol. Homogenates were centrifuged and the supernatant was used for analysis [32]. For each sample, 1 mL of extract was mixed with 1 mL ninhydrin reagent (1% ninhydrin, 60% acetic acid, 20% ethanol, *v*/*v*/*v*) and incubated at 100 °C for 11 min. Reactions were rapidly cooled on ice, and absorbance was measured at 520 nm. Proline concentrations were calculated from an L-proline standard curve and expressed as mg g^−1^ FW. This assay quantifies free proline and was applied consistently across all treatments; therefore, comparisons are valid within the experimental framework of this study.

### 4.7. Antioxidant Enzyme Activities (CAT and POD)

Antioxidant enzyme activities were measured in shoots and roots of seedlings after 21 days of Cd exposure. Approximately 0.25 g fresh tissue was homogenized in 1.0 mL ice-cold 0.1 M potassium phosphate buffer (pH 7.0) containing 1 mM EDTA, 1% (*w*/*v*) polyvinylpyrrolidone (PVP) and 1 mM ascorbic acid. Homogenates were centrifuged at 15,000× *g* for 20 min at 4 °C, and the supernatant was used as the crude enzyme extract.

Total soluble protein was quantified by the Bradford method using bovine serum albumin as standard, and enzyme activities were normalized to protein content (U mg^−1^ protein min^−1^).

Catalase (CAT; EC 1.11.1.6) activity was measured using the ammonium molybdate method. Enzyme extract was incubated with 65 mM H_2_O_2_ in 60 mM phosphate buffer (pH 7.4) at 25 °C for 4 min. The reaction was stopped by adding ammonium molybdate solution to form a yellow molybdate–H_2_O_2_ complex. Absorbance was measured at 405 nm and CAT activity was calculated using an extinction coefficient of 39.4 mM^−1^ cm^−1^. One unit of CAT activity was defined as the amount of enzyme decomposing 1 µmol H_2_O_2_ per minute per mg protein.

Guaiacol peroxidase (POD; EC 1.11.1.7) activity was measured by monitoring the formation of tetraguaiacol. The reaction mixture contained 50 mM phosphate buffer (pH 7.0), 20 mM guaiacol, 20 mM H_2_O_2_ and enzyme extract. After initiating the reaction with H_2_O_2_, the increase in absorbance at 470 nm was recorded for 120 s at 25 °C. Activity was calculated using the molar extinction coefficient for tetraguaiacol (26.6 mM^−1^ cm^−1^) and expressed as µmol tetraguaiacol formed per minute per mg of protein.

All enzyme assays were performed immediately after extraction, and extracts were kept on ice during measurements to minimize changes in enzyme activity.

### 4.8. Mineral Analysis

Elemental concentrations were determined in whole-plant dry biomass collected at the end of the experiment. The dried material was then ground to a fine powder. Approximately 200 mg of powdered tissue was digested in concentrated HNO_3_. Concentrations of Fe, Zn, and Cd were quantified using flame atomic absorption spectrometry (FAAS) with a Varian AA240 FS instrument calibrated against certified standard solutions. Element concentrations were expressed as mg kg^−1^ DW. Elemental analysis was performed using external calibration with certified standard solutions and included procedural blanks and replicate measurements.

Instrument calibration was performed using certified reference standard solutions traceable to the National Institute of Standards and Technology (NIST). Limits of detection (LOD) and quantification (LOQ) were calculated based on the analyte concentrations in method blank samples. For this purpose, a series of independent method blank samples was prepared, and analyte concentrations were measured (Table 10) on a FAAS. The obtained results were used to calculate the limits of detection (LOD) and quantification (LOQ) based on 3σ and 10σ criterion, respectively [35].

The accuracy of the methods applied to complex matrices such as plant material was verified through recovery experiments (spiking). Known quantities of certified analyte standard solutions were added (in increased concentrations) to samples, and the measured concentrations were compared with the theoretically expected values. The recovery factors obtained for the analyzed elements ranged between 92% and 106%, which is consistent with internationally accepted performance criteria for element analysis in environmental and biological matrices [35].

The results presented in Table 9 of the manuscript are all above the established limit of quantification (LOQ) of the method, which serves as the basis for accepting the validity of the analytical results. The capability to reliably measure low cadmium concentrations is confirmed by the method’s limit of detection (LOD) of 0.014 mg/L and limit of quantification (LOQ) of 0.022 mg L^−1^.

### 4.9. RNA Extraction and Transcriptomic Analysis

#### 4.9.1. RNA Extraction and Quality Control

Total RNA was extracted from BP and NP *S. sendtneri* seeds using the NucleoSpin^®^ RNA Plant Kit (Macherey–Nagel, Düren, Germany), following the manufacturer’s protocol with minor modifications optimized for phenolic-rich seed tissue. For each treatment, three biological replicates were prepared, each consisting of pooled seeds from multiple plants.

Approximately 80–100 mg of seeds was frozen in liquid nitrogen and ground to a fine powder in a pre-chilled mortar and pestle. Powdered tissue was transferred to RNase-free tubes and lysed in RA1 buffer supplemented with 1% (*v*/*v*) β-mercaptoethanol. Lysates were clarified through NucleoSpin^®^ filter columns, and RNA was bound to silica membranes under chaotropic conditions. After sequential washing (RA2, RA3 buffers), on-column DNase digestion (rDNase set, Macherey–Nagel) was performed to remove genomic DNA. RNA was eluted in 40–50 µL RNase-free water.

RNA quantity was measured using a Qubit 4 Fluorometer (Thermo Fisher Scientific, Waltham, MA, USA) with the RNA Broad-Range Assay. Purity (A260/A280 and A260/A230) was checked on a NanoDrop spectrophotometer. RNA integrity was evaluated by agarose gel electrophoresis (1% agarose in DEPC-treated buffer) and, for selected samples, by automated electrophoresis (Agilent 2100 Bioanalyzer; Richmond Scientifics, Chorley, UK). Samples with clear 18S and 28S rRNA bands, A260/A280 of 1.9–2.1, A260/A230 > 2.0 and RNA integrity number (RIN) ≥ 6.5 were used for library preparation. RNA was stored at −80 °C until shipment on dry ice for sequencing.

#### 4.9.2. Library Preparation and Sequencing

RNA-seq libraries were prepared by a certified service provider following Illumina standard protocols. Briefly, poly(A)^+^ mRNA was enriched using oligo(dT) magnetic beads and fragmented to ~200–350 bp. First-strand cDNA was synthesized using random hexamer primers and reverse transcriptase, followed by second-strand synthesis with incorporation of dUTP to maintain strand specificity.

Double-stranded cDNA fragments were end-repaired, A-tailed and ligated to indexed Illumina adapters. Adapter-ligated fragments were purified with AMPure XP beads and size-selected. Libraries were amplified by PCR (10–12 cycles), and fragment size distribution (~300–450 bp including adapters) and absence of primer dimers were confirmed using an Agilent Bioanalyzer High Sensitivity DNA assay. Libraries were quantified by qPCR, pooled equimolarly and sequenced on an Illumina NovaSeq 6000 platform using paired-end 150 bp (PE150) chemistry. Each library yielded ~40–55 million read pairs.

#### 4.9.3. De Novo Transcriptome Assembly and Functional Annotation

Raw paired-end reads (FASTQ files) were quality-checked with FastQC and trimmed using Trimmomatic to remove adapters and low-quality bases (SLIDINGWINDOW:4:20, LEADING:3, TRAILING:3, MINLEN:36). Trimmed reads were de novo assembled using Trinity v2.15.2 [36], then the redundancy of transcriptome was reduced by obtaining the longest isoform per gene using the “get_longest_isoform” script included in the software, followed by a run of the algorithm TransDecoder [37], thus resulting predicted proteins were functionally annotated by the use of InterProScan v.5.75.106 [38] and eggNOG-mapper [39] software. The structural and functional annotation was produced using funannotate v1.8.17 [40]. Barrnap v.0.9 [41] and tRNAscan-SE [42] were used for rRNA and tRNA detection, respectively, while remaining unannotated transcripts were evaluated with CPC2 tool [43] to predict potential coding/non-coding probability of them. AGAT tool [44] was used to extract proteins from protein-coding transcripts in FASTA.

#### 4.9.4. Differential Expression Analysis

Following, good quality reads were then aligned back to the *Silene* final transcriptome using Salmon v.1.10.3 [45] with the selective-alignment-based mode for quantify gene expression.

Gene-level read counts were retrieved from the ‘tximport’ package [46] in R environment for statistical analysis, then processed with edgeR software @domenico for TMM normalization before performing the differential gene expression analysis. Genes were considered differentially expressed between BP and NP seeds when |log_2_ fold change| ≥ 1 and adjusted *p*-value (Benjamini–Hochberg FDR) < 0.05.

Functional enrichment analyses of DEGs were performed using String v12.0 [47], accessible as custom organism (https://version-12-0.string-db.org/organism/STRG0A34JKL, accessed on 15 September 2025). For each RNA-seq contrast, transcripts were ranked according to their log2FC, and the up- and down-regulated transcripts selected as input gene set for the analysis. STRING’s built-in enrichment module was used to identify over-represented functional categories, including Gene Ontology (GO) terms for Biological Process (BP), Molecular Function (MF) and Cellular Component (CC), and KEGG pathways based on the KEGG Orthology annotations associated in the custom organism. The enrichment results were retrieved in tab-delimited format, and terms with false discovery rate (FDR)-corrected *p*-values 0.05 were considered significantly enriched.

## 5. Conclusions

This study demonstrates that biopriming *S. sendtneri* seeds with *P. phytofirmans* establishes a stable transcriptomic memory that enhances the plant’s capacity to tolerate and accumulate Cd^2+^. The RNA-seq data show that biopriming activates key pathways linked to metal detoxification, antioxidant defense, osmoprotection, membrane transport and cell-wall reinforcement even before germination, indicating a preparedness state that persists into seedling development.

Physiological and biochemical analyses confirmed that this memory state translates into improved performance under Cd stress. Bioprimed plants maintained higher biomass, chlorophylls and carotenoids, accumulated significantly more proline and sugars, and exhibited markedly stronger antioxidant enzyme activities, especially peroxidases in roots, compared with non-primed plants. Importantly, bioprimed plants preserved essential micronutrient (Fe, Zn) homeostasis while simultaneously showing increased Cd uptake and sequestration, consistent with the early upregulation of ABC transporters, vacuolar exchangers and metal-binding components.

These findings reveal a coordinated priming effect that strengthens both tolerance and hyperaccumulation traits in *S. sendtneri*. The integration of transcriptomic memory with enhanced physiological resilience highlights biopriming as a promising strategy for improving the performance of metal-tolerant species in contaminated environments. This work provides a mechanistic foundation for exploiting PGPR-mediated biopriming to engineer “stress-ready” plants for phytoremediation, ecological restoration and sustainable cultivation on metal-impacted soils. Future studies could extend this work by refining cadmium concentration gradients to further dissect dose-dependent aspects of biopriming-induced transcriptomic memory and stress adaptation in *Silene sendtneri*. Such approaches would complement the present findings by linking memory strength and physiological outcomes across a broader range of Cd^2+^ exposure.

## Figures and Tables

**Table 1:** Summary stats relative to the transcriptome annotation of the 106,576 mRNAs identified in this species.

Database	Gene (Count) Annotated	Percentage of mRNA Covered	Total Assignments	Unique Terms/Groups
Overall Annotation	91,427	86.11%	-	-
EggNOG	90,787	85.51%	90,787	17,253
COG	85,374	80.41%	91,138	24 (categories)
PFAM	5994	56.46%	106,366	5138
InterPro	16,905	15.92%	46,591	657
BUSCO	3201	3.01%	3205	122

**Table 2:** Examples of upregulated DEGs (BP vs. NP).

Gene ID	log2FC	padj	Annotation
Cluster-18721.87305	+12.10	3.7 × 10^−20^	Calcium uptake protein 1 homolog
Cluster-18721.139748	+10.50	6.4 × 10^−16^	ABC transporter C family 14-like
Cluster-18721.92466	+10.39	1.0 × 10^−15^	Profilin-2-like
Cluster-18721.92854	+9.16	9.3 × 10^−16^	Chloroplastic glutathione reductase-like
Cluster-18721.198570	+9.02	2.6 × 10^−15^	Detoxification 27-like peroxidase
Cluster-18721.92406	+8.88	5.0 × 10^−14^	RBOH-like NADPH oxidase
Cluster-18721.99315	+8.74	1.9 × 10^−13^	Heat shock protein, chloroplastic
Cluster-18721.96314	+8.41	4.1 × 10^−13^	Glycosyltransferase family protein
Cluster-18721.207948	+8.02	1.2 × 10^−12^	ABC transporter G family 24-like
Cluster-18721.149205	+7.51	4.2 × 10^−12^	Vacuolar cation/proton exchanger 2 (CAX2-like)
Cluster-18721.147169	+7.41	8.1 × 10^−12^	Zinc transporter 4
Cluster-18721.94369	+7.12	3.3 × 10^−11^	Light-harvesting chlorophyll a/b protein
Cluster-18721.92883	+6.98	5.9 × 10^−11^	Glycosyltransferase family 14
Cluster-18721.144837	+6.81	1.4 × 10^−10^	Delta-1-pyrroline-5-carboxylate synthetase (P5CS)
Cluster-18721.95041	+6.45	3.7 × 10^−10^	Phenylpropanoid pathway O-methyltransferase
Cluster-18721.108228	+6.37	7.9 × 10^−10^	MEROPS metalloprotease-like
Cluster-18721.90900	+6.32	1.4 × 10^−9^	ABC transporter G family 31-like
Cluster-18721.103354	+6.16	2.1 × 10^−9^	Chlorophyll *a*/*b*-binding protein CP26-like
Cluster-18721.108954	+6.12	3.0 × 10^−9^	Heat shock protein 20 (chloroplastic)
Cluster-18721.78843	+5.92	3.8 × 10^−9^	UDP-glycosyltransferase-like
Cluster-18721.86696	+5.77	6.1 × 10^−9^	WRKY transcription factor-like
Cluster-18721.145987	+5.51	1.1 × 10^−8^	Cytochrome P450 reductase
Cluster-18721.78811	+5.43	3.0 × 10^−8^	Lignin biosynthesis peroxidase

**Table 3:** Examples downregulated DEGs (BP vs. NP).

Gene ID	log2FC	padj	Annotation
Cluster-18721.122004	−9.12	2.2 × 10^−11^	Magnesium transporter MRS2-4-like
Cluster-18721.129884	−8.91	4.9 × 10^−11^	Magnesium transporter NIPA9
Cluster-18721.104214	−8.43	1.2 × 10^−10^	NRAMP3-like metal transporter
Cluster-18721.131048	−7.98	4.1 × 10^−10^	NRT2.5-like nitrate transporter
Cluster-18721.128000	−7.52	6.9 × 10^−10^	Proline-rich cell-wall protein
Cluster-18721.140001	−7.18	2.8 × 10^−9^	Photosystem I reaction center subunit F
Cluster-18721.160094	−7.01	4.9 × 10^−9^	RNA-binding protein-like
Cluster-18721.119954	−6.62	1.2 × 10^−8^	Glutathione peroxidase (isoform 2)
Cluster-18721.90512	−6.55	1.9 × 10^−8^	Vacuolar iron transporter-like
Cluster-18721.140005	−6.32	3.0 × 10^−8^	Major facilitator superfamily (MFS) transporter
Cluster-18721.162512	−6.15	4.7 × 10^−8^	Pectinesterase inhibitor-like
Cluster-18721.107779	−5.83	7.3 × 10^−8^	WRKY-like transcription factor (repressed isoform)
Cluster-18721.112993	−5.74	1.1 × 10^−7^	Peroxidase P7-like (isoform downregulated)
Cluster-18721.121443	−5.31	1.9 × 10^−7^	Serine/threonine kinase-like
Cluster-18721.95314	−5.11	3.2 × 10^−7^	Amino acid permease AAP6-like
Cluster-18721.90143	−5.06	5.0 × 10^−7^	Ubiquitin-protein ligase-like
Cluster-18721.15400	−4.91	1.2 × 10^−6^	Cytochrome P450 family protein (repressed isoform)
Cluster-18721.90063	−4.65	2.4 × 10^−6^	Aquaporin PIP2-7-like
Cluster-18721.90832	−4.47	3.3 × 10^−6^	Nucleoside transporter-like
Cluster-18721.99481	−4.31	8.8 × 10^−6^	Glutamate synthase-like

**Table 4:** Functionally relevant DEGs by category.

Category	Regulation	Key Genes (Examples)	Biological Role
Antioxidant/ROS detox	up	Glutathione reductase, Detox27 peroxidase, RBOH-like	GSH cycling, H_2_O_2_ detox, redox buffering
	down	GPX-like, peroxidase P7-like	Isoform rebalancing
Metal transport & sequestration	up	ABC transporters C14 & G24/31, CAX2, ZnT1/4	Vacuolar Cd^2+^ sequestration, micronutrient stabilization
	down	NRAMP3, Mg transporters (NIPA9, MRS2)	Reduced metal remobilization
Osmoprotectants	up	P5CS, glycosyltransferases	Proline biosynthesis, sugar accumulation
Photosynthesis & pigments	up	LHC proteins (CP26), HSP20-Chl	Stabilization of chlorophyll complexes
	down	PSI reaction center F	Fine-tuning of photosynthetic electron flow
Cell wall & phenylpropanoids	up	COMT, lignin peroxidases	Cell-wall thickening, Cd^2+^ immobilization
Stress signaling & transcription	up	WRKY, MYB, NAC, MAPK-associated genes	transcriptional regulation, early defense activation
	down	WRKY isoform (repressed), receptor kinases	Repressor suppression

**Table 5:** Biomass in nonprimed (NP) and bioprimed (BP) *S. sendtneri* under Cd^2+^ stress.

Treatment	Cd^2+^ (mM)	FW Shoot (mg)	DW Shoot (mg)	FW Root (mg)	DW Root (mg)
NP	0	11.26 ± 0.02 ^c^	1.23 ± 0.04 ^b^	1.04 ± 0.04 ^c^	0.24 ± 0.07 ^d^
NP	0.25	3.17 ± 0.02 ^d^	0.94 ± 0.08 ^c^	0.98 ± 0.02 ^d^	0.17 ± 0.05 ^d^
NP	0.50	–	–	–	–
BP	0	12.59 ± 0.06 ^b^	2.05 ± 0.03 ^a^	5.19 ± 0.07 ^a^	0.74 ± 0.09 ^a^
BP	0.25	15.62 ± 0.08 ^a^	2.06 ± 0.03 ^a^	2.32 ± 0.03 ^b^	0.52 ± 0.01 ^b^
BP	0.50	11.91 ± 0.02 ^c^	0.77 ± 0.02 ^d^	1.34 ± 0.01 ^b^	0.34 ± 0.02 ^c^

NP on 0.5 mM Cd^2+^ did not fully develop and was excluded from further analysis. Treatments sharing same letter within one column do not differ significantly after Newman Keuls ANOVA post hoc test at level of significance *p* < 0.05.

**Table 6:** Photosynthetic pigments in nonprimed (NP) and bioprimed (BP) *S. sendtneri* under Cd^2+^ stress.

Treatment	Cd^2+^ (mM)	Chlorophyll *a* mg g^−1^ DW	Chlorophyll *b* mg g^−1^ DW	Carotenoids mg g^−1^ DW
NP	0	12.50 ± 0.11 ^c^	5.70 ± 0.05 ^b^	3.25 ± 0.00 ^c^
NP	0.25	1.40 ± 0.02 ^e^	1.96 ± 0.05 ^d^	1.15 ± 0.01 ^e^
NP	0.50	-	-	-
BP	0	14.48 ± 0.01 ^b^	6.92 ± 0.05 ^a^	5.28 ± 0.01 ^a^
BP	0.25	15.11 ± 0.02 ^a^	6.39 ± 0.01 ^a^	4.94 ± 0.01 ^b^
BP	0.50	9.04 ± 0.01 ^d^	3.59 ± 0.01 ^c^	2.83 ± 0.01 ^d^

NP on 0.5 mM Cd^2+^ did not fully develop and was excluded from further analysis. Treatments sharing same letter within one column do not differ significantly after Newman Keuls ANOVA post hoc test at level of significance *p* < 0.05.

**Table 7:** Proline content in nonprimed (NP) and bioprimed (BP) *S. sendtneri* under cadmium stress.

Treatment	Cd^2+^ (μM)	Proline—Shoot (mg g^−1^ FW)	Proline—Root (mg g^−1^ FW)
NP	0	5.37 ± 0.01 ^c^	2.41 ± 0.02 ^a^
NP	0.25	6.96 ± 0.07 ^c^	2.51 ± 0.05 ^a^
NP	0.50	-	-
BP	0	14.49 ± 0.11 ^a^	0.23 ± 0.01 ^c^
BP	0.25	15.11 ± 0.02 ^a^	1.26 ± 0.03 ^b^
BP	0.50	9.04 ± 0.02 ^b^	1.27 ± 0.01 ^b^

NP on 0.5 mM Cd^2+^ did not fully develop and was excluded from further analysis. Treatments sharing same letter within one column do not differ significantly after Newman Keuls ANOVA post hoc test at level of significance *p* < 0.05.

**Table 8:** Antioxidant enzyme activity (CAT and POD) in shoots and roots of nonprimed (NP) and bioprimed (BP) *S. sendtneri* under Cd^2+^ stress.

Treatment	Cd^2+^ (mM)	CAT Shoot	CAT Root	POD Shoot	POD Root
NP	0	18.16 ± 0.44 ^b^	7.88 ± 0.34 ^b^	8.16 ± 0.44 ^c^	1.88 ± 0.34 ^d^
NP	0.25	37.24 ± 1.86 ^a^	37.65 ± 1.44 ^a^	7.24 ± 0.16 ^c^	7.65 ± 0.44 ^d^
NP	0.50	-	-	-	-
BP	0	1.04 ± 0.08 ^d^	0.26 ± 0.02 ^c^	423.67 ± 20.19 ^a^	3020.036 ± 142.78 ^a^
BP	0.25	4.29 ± 0.07 ^c^	1.04 ± 0.06 ^c^	174.62 ± 30.79 ^b^	6435.34 ± 181.04 ^b^
BP	0.50	0.89 ± 0.03 ^d^	0.23 ± 0.01 ^c^	129.73 ± 26.14 ^b^	727.38 ± 17.80 ^c^

NP on 0.5 mM Cd^2+^ did not fully develop and was excluded from further analysis. All values in UNITS mg^−1^ protein min^−1^, mean ± SD. Treatments sharing same letter within one column do not differ significantly after Newman Keuls ANOVA post hoc test at level of significance *p* < 0.05.

**Table 9:** Concentrations of Fe, Cd, and Zn determined in whole-plant dry biomass of non-primed (NP) and bioprimed (BP) *S. sendtneri* grown under control conditions and in the presence of 0.25 mM and 0.5 mM Cd.

Treatment	Cd^2+^ (mM)	Fe (mg kg^−1^ DW)	Cd (mg kg^−1^ DW)	Zn (mg kg^−1^ DW)
NP	0	208.43 ± 1.80 ^c^	1.11 ± 0.18 ^d^	45.45 ± 0.60 ^d^
NP	0.25	67.28 ± 1.02 ^e^	100.41 ± 0.82 ^c^	14.02 ± 2.96 ^e^
NP	0.50	–	–	–
BP	0	444.28 ± 4.20 ^b^	7.53 ± 0.30 ^d^	112.95 ± 1.00 ^b^
BP	0.25	197.52 ± 11.39 ^d^	854.46 ± 2.97 ^b^	153.32 ± 6.09 ^a^
BP	0.50	1596.15 ± 23.08 ^a^	1412.50 ± 10.96 ^a^	57.26 ± 7.26 ^c^

NP on 0.5 mM Cd^2+^ did not fully develop and was excluded from further analysis. Values are mean ± SD. Treatments sharing same letter within one column do not differ significantly after Newman Keuls ANOVA post hoc test at level of significance *p* < 0.05.

**Table 10:** Analytical quality control parameters for Cd^2+^, Fe, and Zn.

Recovery Results for Elements in Spiked Samples					
Element	Sample (mg L^−1^)	Spike	Determined	R%	$\bar{R}\%$
Cd	1.50	0.25	1.73	92	94
		0.50	1.97	94	
		2.00	4.44	97	
Fe	0.484	0.25	0.740	102	104
		0.50	1.002	103	
		2.00	2.604	106	
Zn	0.374	0.25	0.610	94	96
		0.50	0.862	97	
		2.00	2.326	97	
**Method blank concentrations (mg L^−1^) and analytical performance parameters (LOD and LOQ) for Cd, Fe, and Zn**					
Cd	C, method blank (mg L^−1^)	Fe	C, method blank (mg L^−1^)	Zn	C, method blank (mg L^−1^)
	0.012		0.034		0.008
	0.010		0.020		0.012
	0.009		0.028		0.015
	0.009		0.030		0.009
	0.011		0.025		0.011
	0.012		0.034		0.008
	0.010		0.020		0.012
	0.009		0.028		0.015
	0.009		0.030		0.009
	0.011		0.025		0.011
	LOD = 0.014		LOD = 0.042		LOD = 0.019
	LOQ = 0.022		LOQ = 0.077		LOQ = 0.037

Spike = added concentration; R%, Recovery factor = [(Determined − Sample)/Spike] × 100); $\bar{R}\%$ = mean. Recovery factor; LOD = Limit of Detection (3σ criterion); LOQ = Limit of Quantification (10σ criterion).

## Data Availability

Full list of up- and downregulated genes available upon request.

## Supplementary Materials

The following supporting information can be downloaded at: https://www.mdpi.com/article/10.3390/plants15020257/s1, Table S1. Targeted analysis of gene families and functional modules relevant to cadmium tolerance in bioprimed (BP) vs. non-primed (NP) *S. sendtneri* seeds (RNA-seq).
