# Supplementary material for: Biopriming-Induced Transcriptomic Memory Enhances Cadmium Tolerance in the Cd Hyperaccumulator Silene sendtneri
Source: Plants (Basel). 2026 Jan 14;15(2):257. doi: 10.3390/plants15020257 (PMC12844900; doi:10.3390/plants15020257)
Supplement: Supplementary file 1 [file plants-15-00257-s001.zip › plants-4051162-supplementary.pdf]

Supplementary table S1

argeted analysis of gene families and functional modules relevant to cadmium tolerance in bioprimed (BP) vs non-primed (NP) S. sendtneri seeds (RNA-seq).

Functional module (Cd tolerance)	Representative DEGs (Bioprimed vs Nonprimed) from this study	Direction of expression in biprimed plants	Mechanistic relevance
**Thiol/redox maintenance (glutathione-linked)**	Chloroplastic glutathione reductase-like (Cluster-18721.92854; log2FC +9.16)	↑	Maintains reduced glutathione pool; supports thiol-dependent detoxification capacity and redox buffering
**ROS signalling & scavenging**	Detoxification 27-like peroxidase (Cluster-18721.198570; +9.02); Class III peroxidases (e.g., lignin biosynthesis peroxidase, Cluster-18721.78811; +5.43); RBOH-like NADPH oxidase (Cluster-18721.92406; +8.88)	↑	Peroxidase-centered ROS detoxification; regulated ROS signalling via RBOH; limits oxidative damage under metal stress
**Metal transport & vacuolar sequestration**	ABC transporter C family 14-like (Cluster-18721.139748; +10.50); ABC transporter G family 24/31-like (Cluster-18721.207948; +8.02; Cluster-18721.90900; +6.32); CAX2-like exchanger (Cluster-18721.149205; +7.51)	↑	Transport and vacuolar compartmentalization of metal complexes; enhanced sequestration capacity
**Micronutrient transport rebalancing**	Zinc transporter 4 (Cluster-18721.147169; +7.41); NRAMP3-like metal transporter (Cluster-18721.104214; −8.43); Vacuolar iron transporter-like (Cluster-18721.90512; −6.55)	↑ / ↓	Stabilizes micronutrient homeostasis under Cd competition; reduces remobilization routes associated with displacement
**Osmoprotection / compatible solutes**	P5CS (Cluster-18721.144837; +6.81); Glycosyltransferase family proteins (Cluster-18721.96314; +8.41;	↑	Supports proline synthesis and carbohydrate remodelling linked to stress tolerance and osmoprotective capacity

	Cluster-18721.92883; +6.98)		
**Cell wall / phenylpropanoid reinforcement**	Phenylpropanoid O-methyltransferase (Cluster-18721.95041; +6.45); Lignin biosynthesis peroxidase (Cluster-18721.78811; +5.43)	↑	Cell wall strengthening; structural restriction/immobilization of metals and reinforcement under stress
**Transcriptional regulation (stress response)**	WRKY TF-like (Cluster-18721.86696; +5.77); WRKY-like TF (repressed isoform) (Cluster-18721.107779; −5.83)	↑ / ↓	Regulatory reprogramming consistent with priming-induced transcriptional state; isoform-level balancing
**Plastid protection / protein stability**	HSP20 (chloroplastic) (Cluster-18721.108954; +6.12); chloroplastic HSP (Cluster-18721.99315; +8.74); LHC a/b proteins (Cluster-18721.94369; +7.12; Cluster-18721.103354; +6.16)	↑	Stress-associated protection of photosynthetic machinery; consistent with pigment stability observed under Cd
